# Making the Environmental Justice Grade: The Relative Burden of Air Pollution Exposure in the United States

**DOI:** 10.3390/ijerph8061755

**Published:** 2011-05-25

**Authors:** Marie Lynn Miranda, Sharon E. Edwards, Martha H. Keating, Christopher J. Paul

**Affiliations:** 1 Children’s Environmental Health Initiative, Nicholas School of the Environment, Duke University, Box 90328, Durham, NC 27708, USA; E-Mails: se@duke.edu (S.E.E.); christopher.paul@duke.edu (C.J.P.); 2 Keating Environmental, 7508 Thunder Mountain, Efland, NC 27243, USA; E-Mail: martha.keating@gmail.com

**Keywords:** environmental justice, air pollution, ozone, particulate matter

## Abstract

This paper assesses whether the Clean Air Act and its Amendments have been equally successful in ensuring the right to healthful air quality in both advantaged and disadvantaged communities in the United States. Using a method to rank air quality established by the American Lung Association in its 2009 State of the Air report along with EPA air quality data, we assess the environmental justice dimensions of air pollution exposure and access to air quality information in the United States. We focus on the race, age, and poverty demographics of communities with differing levels of ozone and particulate matter exposure, as well as communities with and without air quality information. Focusing on PM_2.5_ and ozone, we find that within areas covered by the monitoring networks, non-Hispanic blacks are consistently overrepresented in communities with the poorest air quality. The results for older and younger age as well as poverty vary by the pollution metric under consideration. Rural areas are typically outside the bounds of air quality monitoring networks leaving large segments of the population without information about their ambient air quality. These results suggest that substantial areas of the United States lack monitoring data, and among areas where monitoring data are available, low income and minority communities tend to experience higher ambient pollution levels.

## 1. Introduction

The Clean Air Act of 1970, as amended in 1990, gives the United States Environmental Protection Agency (EPA) the mandate to regulate air pollutant emissions. Under the Clean Air Act, the EPA is required to set National Ambient Air Quality Standards (NAAQS) for pollutants considered harmful to public health and the environment within an “adequate margin of safety” [1]. In response to this mandate, the EPA has established NAAQS for six major pollutants, most commonly called the “criteria air pollutants,” including particulate matter (PM_10_ and PM_2.5_) and ozone. In setting NAAQS, the EPA explicitly considers sensitive subpopulations [1]. Because the Clean Air Act establishes an expectation of clean air everywhere through nationally uniform standards, it essentially establishes clean air as a right of all people of the United States. This paper explores whether differential potential exposure to ozone and particulate matter exist between advantaged and disadvantaged populations.

Significant research has been directed toward understanding the potential health effects of air pollution. After major improvements in air quality in the 1970s and 1980s, research now focuses on the impacts of lower level chronic exposure to various pollutants, including fine particulate matter (PM_2.5_) and ozone [2]. Fine particles penetrate deeply into the respiratory system and may have other toxic substances (lead, sulfates, and various metals) adsorbed to their surface [2]. Even at levels lower than the current NAAQS, particulate matter and ozone are linked to mortality and hospital visits [3,4], commonly through their impact on respiratory and cardiovascular disease [2,5]. In addition, exposure to particulate matter and ozone has been linked to poor birth outcomes [6–9].

To assess whether geographic areas are in compliance with the NAAQS, a network of air pollution monitors that measure ambient levels of each of the criteria pollutants has been established across the United States. The number of monitors in a given location typically reflects the population density of the area with a minimum number of monitors prescribed by regulation. For ozone, a minimum of two monitors are required for areas with a population greater than 200,000. If an area has an ozone nonattainment designation of serious, severe, or extreme, up to five monitors are required [10]. For PM_2.5_, the number of required monitors ranges from 1 in metropolitan statistical areas (MSAs) with populations greater than 200,000, to 10 in MSAs with populations greater than 8 million [11]. The monitoring network used for ozone is separate from and uses different equipment than the monitoring network for particulate matter.

The principal objective of the monitoring network is to measure ambient concentrations of various pollutants where people live, work, and play. Ozone monitors are placed to measure the ozone concentration where the highest population density might be exposed to a significant ozone concentration and in areas with maximum downwind concentration [10]. For compliance with the annual PM_2.5,_ standard, monitors are located at sites that represent exposure on an urban or community scale, while sites representing maximum exposure are selected for evaluation against the short-term 24-hour standard [11].

The American Lung Association (ALA) issues an annual report entitled “The State of the Air,” which uses data from the U.S. EPA’s Air Quality System (AQS) to characterize the ozone and PM_2.5_ concentrations at each monitoring site across the United States [12]. These two pollutant are especially relevant for evaluating human health impacts, as they have been linked to adverse health outcomes, even at low concentration levels [2,6]. The ALA describes trends in the number of sites where air quality has either improved or worsened over the past year and lists America’s “cleanest” and “dirtiest” cities with respect to air quality.

This paper uses the air quality ranking approach established by the ALA in its 2009 State of the Air report along with EPA air quality data to assess the environmental justice (EJ) dimensions of air pollution exposure in the United States. The EPA defines environmental justice as:

“the fair treatment and meaningful involvement of all people regardless of race, color, national origin, or income with respect to the development, implementation, and enforcement of environmental laws, regulations, and policies. EPA has this goal for all communities and persons across this Nation [13].”

Racial and economic disparity in environmental quality have long been recognized in the United States. The environmental justice movement arose from community concern that toxic waste sites were disproportionally located in poor and minority communities, as described by Robert Bullard in his early work, including “Dumping in Dixie” [14]. A 1987 report by the United Church of Christ Commission for Racial Justice, “Toxic Wastes and Race in the United States” catalyzed national attention when it documented that hazardous waste facilities were sited disproportionately in communities of color [15]. This report was updated in 2007 to use 2000 Census data and more sophisticated spatial analytical techniques, and found that disparities in the location of hazardous waste sites has persisted [16].

Research on environmental justice and equity has investigated whether certain groups have higher exposure to pollution, lower overall environmental quality and amenities, and abnormally high rates of environmentally-driven disease compared to other racial, ethnic, or socioeconomic groups. These studies have been conducted on a variety of pollutants and pollution sources including air pollution, Superfund sites, and manufacturing facilities [17–20]. These analyses have generally demonstrated a correlation with both income and race though there is much variation across regions and amongst these variables that is not well explained [19,21]. Spatially-based approaches are powerful ways to characterize environmental inequity [22,23].

In this paper, we focus on the EJ implications of poor air quality and of monitoring network design by analyzing the race, ethnicity, age, and poverty demographics of communities with differing levels of ozone and particulate matter exposure. The objectives of this paper are to:

- Determine whether counties with sufficient AQS monitoring data are different on key demographic variables compared to those without AQS monitors or with insufficient data (this speaks to the location of the AQS monitors).
- Use the ALA methodology for rating air quality to conduct a national county-level analysis assessing the association between air quality and race, ethnicity, age, and poverty rates.
- Use a buffer analysis to develop a highly resolved geographic analysis of the EJ implications of air quality in the United States.

We are particularly interested in how the implementation of the Clean Air Act and its Amendments has shaped air quality in both advantaged and disadvantaged communities in the United States.

## 2. Experimental Section

### 2.1. Air Quality Monitoring Data

We queried the AQS Data Mart [24] for daily 8-hr maximum concentrations of ozone at each monitoring site in the United States in 2005, 2006, and 2007. Consistent with the methodology employed by the ALA, only measurements during the EPA-defined ozone season and only 8-hr ozone concentrations that were based on 6–8 hours of hourly measurements were included in the analysis. The ozone season is designated by EPA on a state-by-state basis, reflecting different seasons of hot weather. Most states in the contiguous United States have an April to September–October ozone season [25].

Daily 24-hour averaged PM_2.5_ concentrations collected using EPA approved reference and equivalent methods during the 2005–2007 period at each monitoring site were also obtained from the AQS database [26]. Annual PM_2.5_ data for 2005–2007 were downloaded as EPA-summarized, annual design values at the county-level [27].

### 2.2. From Monitoring Data to Air Quality Metrics

We reproduced the ranking methodology for ozone and daily PM_2.5_ previously described in detail in the ALA State of the Air 2009 report [12]. We specifically chose to use the ALA approach because it is highly-regarded and widely cited. The annual report presents a practical and transparent way to condense and present thousands of data points in a manner that is understandable and familiar to the general public and to policymakers. The ALA assigns grades separately for both ozone levels and daily PM_2.5_ levels ranging from “A” to “F” to each county in the United States (where sufficient monitoring data are available for each pollutant). These grades are based on the weighted average of the number of days on which at least one monitor in a county reported a pollution level reaching an air quality designation of orange (unhealthy for sensitive populations, weighting factor = 1), red (unhealthy, weighting factor = 1.5), purple (very unhealthy, weighting factor = 2), or maroon (hazardous, weighting factor = 2.5) [12]. The ALA uses the county-level weighted average number of poor air quality days to assign a letter grade to each county based on the severity of air pollution. This grading was done separately for ozone and daily PM_2.5_ both because the two pollutants are measured by different monitors which may or may not be placed in the same counties, but also because they represent different reasons for concern. Our methodology differed from that of the ALA in that we did not use the letter grade categorization, but instead used the weighted average of the number of days with poor air quality as a direct metric of air quality. In addition to the county-level analysis, we used the same approach to calculate weighted average number of poor air quality days for each individual ozone and daily PM_2.5_ monitor, allowing us to do sub-county analysis.

In addition to metrics based on daily ozone and PM_2.5_ data, attainment of the year-round particulate pollution (annual PM_2.5_) NAAQS is accessed using “design values” that are calculated by EPA. A design value is defined by EPA as a statistic, based on multiple years of monitoring data which describes the air quality status of a given area relative to the level of the NAAQS [28]. In our analysis, we used the 2005–2007 annual PM_2.5_ design values to rank county-level air quality. Since annual PM_2.5_ design values are available only at the county-level, individual monitoring sites could not be ranked for annual PM_2.5_. Note that the daily ozone and PM_2.5_ metrics provide measures of short term peak exposures, and the annual PM_2.5_ design value metric provides a measure of long term exposure.

We used three air quality metrics—weighted averages of the number of poor air quality days for (1) daily ozone, (2) daily PM_2.5_, and (3) design values for annual PM_2.5_—to rank order all counties with sufficient air quality monitoring data. This allowed us to identify the 20% of counties with the best air quality and the 20% of counties with the worst air quality for each pollution metric. (We note that other comparisons are certainly available and relevant; we chose the top/bottom 20% because it allows us to compare extremes while maintaining a sufficiently large sample size—and is also a familiar metric comparison to the general public.) We first determined the best 20% and worst 20% of counties nationally and then did the same for each of the individual 10 EPA regions. Then, for ozone and daily PM_2.5_, we ranked individual monitoring sites based on the weighted averages of the number of poor air quality days to identify the 20% of monitoring sites with the best and worst air quality. The monitor-level rankings were only done at the national level.

### 2.3. Demographic Data

Demographic and socioeconomic data were obtained from the 2000 U.S. Census. County and block group level data on age, race, ethnicity, and poverty status were extracted from the Summary Tape File 3 database [29]. Using standard variable definitions from the U.S. Census, we identified percent under 5 years of age, percent 65 years of age and older, percent non-Hispanic black (NHB), percent Hispanic, and percent in poverty as key metrics of environmental justice. While many other demographic variables have been considered in the literature (e.g., percent female-headed households [19]), our selected variables are highly correlated with those of other studies. Recognizing that 2000 Census data may now be outdated and not fully reflect the current composition of communities, we explored using the U.S. Census 2005–2007 American Community Survey (ACS) estimates of key demographic and socioeconomic measures. Although the ACS estimates temporally correspond with the air quality data used in this analysis, ACS data is currently limited to geographic areas with at least 20,000 people [30]. Given the limitations of the ACS, we believe that the advantages of using the Census 2000 data, including the ability to use more geographically refined data and the inclusion of geographic areas with fewer than 20,000 people, outweighed the issue of using older demographic and socioeconomic data in this case. The Census 2000 data will still highlight any systematic differences in the populations affected by different levels of air pollution, and once the 2010 U.S. Census data are available, we plan to run the analyses presented here with updated air quality and demographic data.

### 2.4. Statistical Analysis

For counties without air quality monitoring data, or less than three years of monitoring data in the 2005–2007 study period, we could not calculate the air quality metrics. In order to determine if there were differences between communities with access to information about their air quality and those lacking such access or information, we compared the Census 2000 county-level rates of key demographic and socioeconomic indicators for counties with and without monitoring data using population-weighted univariate t-tests. The demographic composition of the communities in monitored *versus* unmonitored counties was compared at both the national and EPA regional level. Because ozone and PM_2.5_ have separate monitoring networks and the data requirements differ between the ALA weighted average number of poor air quality days for short-term PM_2.5_ metric and the EPA-calculated annual design values, the demographic comparisons constitute three separate analyses.

Among those counties with monitoring data in all three years, we compared the communities in the 20% of counties with the best air quality to communities in the 20% of counties with the worst air quality. In order to determine if there were demographic differences between communities with the best and worst air quality, we compared the Census 2000 county-level rates of key demographic and socioeconomic indicators using population-weighted univariate t-tests and multivariate logistic regression controlling for county population. The demographic composition of the communities in best *versus* worst counties was compared across the entire United States and within each EPA region. Because ozone, daily PM_2.5_, and annual PM_2.5_ all have separate monitoring networks, the demographic comparisons constitute three separate analyses.

In addition to county-level analysis, we conducted a more spatially-refined buffer analysis. For monitoring sites active each year from 2005–2007, a site-level weighted average of poor air quality days was calculated for ozone and daily PM_2.5_ per ALA methodology. Site-specific data were not available for annual PM_2.5_, so this air quality metric could not be included in this aspect of the analysis. Monitoring sites were georeferenced using latitude and longitude coordinates provided with the AQS data and a 5 km buffer was constructed around each site. A 5 km buffer was selected to represent a neighborhood scale assessment consistent with dispersion characteristics of PM and ozone. Of note, we constructed 10 and 15 kilometer buffers and obtained similar results. Using the 50% areal containment method [31], in which a geographic unit is considered fully within the buffer zone if at least 50% of the unit’s area is captured by the buffer, we identified those Census 2000 block groups within the buffer zone of monitoring sites ranked as being among the 20% of sites with the best or worst air quality. Multiple methods for constructing buffers (e.g., buffer and clip, Census areal unit centroids [32]) exist with no demonstrated superiority of any one method. Similar to the above analysis, population-weighted univariate t-tests and multivariate logistic regression controlling for population within the buffer were used to determine if the rates of the selected demographic and socioeconomic indicators differed between communities around monitoring sites with the best and worst air quality across the United States.

In summary, in all counties in the U.S. as a whole and disaggregated by EPA region, we compared the demographics of monitored *versus* unmonitored counties. In addition, among counties with monitoring data, we compared the demographics of the best and worst air quality counties. To explore the potential importance of geographic scale, we also created buffers around the best and worst monitors (in terms of air quality) across the United States and then compared the demographics within the buffers.

All statistical analysis was undertaken in SAS 9.2 (SAS Institute, Cary, NC, USA). Statistical tests were conducted with an *α* of 0.05. Since we conducted a large number of weighted t-tests within each set of analyses, we controlled for multiple comparisons by applying Bonferroni’s correction within each set of analyses (*i.e.*, county-level national and regional monitored *versus* unmonitored analysis, county-level national and regional cleanest *versus* dirtiest, and buffer-level national cleanest *versus* dirtiest).

## 3. Results and Discussion

### 3.1. Demographic Differences in Communities with and without Air Quality Data

**Figure 1.**
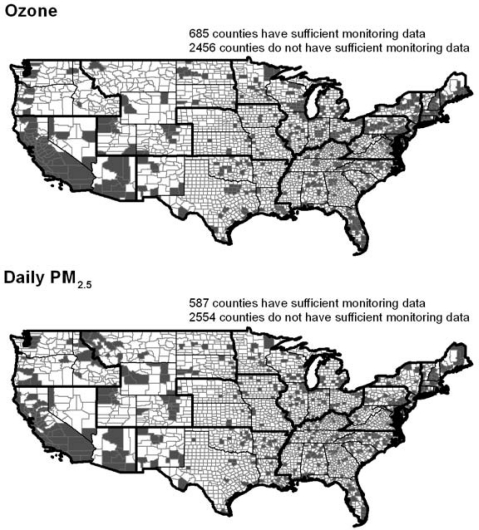

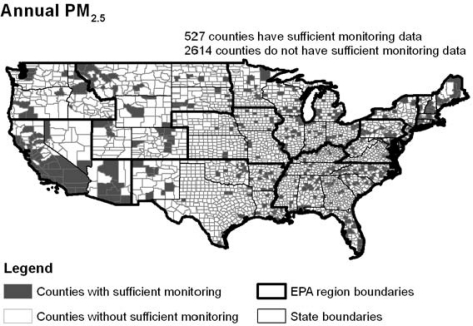
County with and without sufficient air quality monitoring in 2005, 2006, and 2007 to calculate each air quality metric.

Air pollution metrics were calculated for each county with sufficient monitoring data for ozone, daily PM_2.5_, and annual PM_2.5_ based on the methodology established by the ALA in the 2009 State of the Air report. For ozone and daily PM_2.5_, counties with monitoring data for a particular pollutant in 2005, 2006, and 2007 had sufficient data to apply the ALA methodology, while counties without monitors or with data missing for any or all of the 3 years did not have sufficient monitoring data. For annual PM_2.5_, counties without monitors or without enough monitoring values in each year 2005–2007 for the EPA to calculate a design value were considered not to have sufficient monitoring data. The maps in Figure 1 show the geographic distribution of counties with and without sufficient monitoring data to calculate air pollution metrics. The demographic characteristics of counties with sufficient air quality monitoring data were compared to counties with insufficient or no air quality monitoring data. Table 1 presents the results of this analysis in all counties in the U.S. as a whole and disaggregated by EPA region. In this analysis, U.S. territories were excluded, thus adjusting the geographic coverage of Region 2 (excluded Puerto Rico and the U.S. Virgin Islands) and Region 9 (excluded Guam, Trust Territories, American Samoa, and the Northern Mariana Islands).

Of the 3,141 counties in the United States, 527 had sufficient monitoring data for annual PM_2.5_, 587 had sufficient monitoring data for daily PM_2.5_, and 685 had sufficient monitoring data for ozone. As Table 1 demonstrates, there are clear differences in the demographic characteristics of counties with sufficient air quality data to allow calculation of an air quality metric. At the national level, counties without air quality metrics for annual PM_2.5_, daily PM_2.5_, and ozone are characterized by a lower percent NHB, lower percent Hispanic, lower percent under 5 years of age, and higher percent 65 years and older. Using population-weighted t-tests and the Bonferroni correction for multiple comparisons, almost all of these differences were significant at an overall α of 0.05. Counties without air quality metrics for annual PM_2.5_ and daily PM_2.5_ exhibit no differences in percent in poverty; however, counties without air quality metrics for ozone are characterized by a higher percent in poverty. These patterns generally held within the 10 EPA regions, although statistical significance under the correction for multiple comparisons varied.

**Figure 2.**
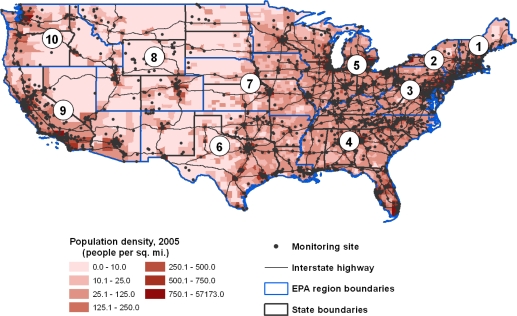
2005 United States county-level population density and EPA regions, overlaid with ozone and PM2.5 air quality monitoring sites.

The observed differences in the demographic characteristics of areas for which we could and could not calculate the 3 air quality metrics likely result from the strategies that the EPA pursues in siting monitors. The map in Figure 2 makes it clear that monitoring efforts for ozone and particulate matter are targeted at areas with high population density and along major interstate highways or heavily industrialized areas. While this system for placing air quality monitors captures data where the highest population density is expected to experience significant ozone and PM_2.5_ exposure, it leaves rural areas with generally older, non-Hispanic white populations with limited air quality data.

### 3.2. County Demographics and Air Quality

Among those counties with sufficient data to calculate an air quality metric (see Figure 1), we compared communities with the most extreme air quality based on each pollution metric. Specifically, we employed univariate analysis using population-weighted t-tests to compare the demographic characteristics of the 20% of counties with the best air quality with the 20% of counties with the worst air quality. For all three pollution metrics, the proportion of NHB in those counties with the worst air quality is over twice the corresponding proportion in those counties with the best air quality (significant at α = 0.05 with Bonferroni correction). A higher percent Hispanic, higher percent under 5 years of age, and lower percent 65 years and older are characteristic of counties in the worst 20% rather than best 20% of ozone-graded counties (p significant at α = 0.05 with Bonferroni correction). For both annual and daily PM_2.5_, counties with the worst air quality have higher rates of poverty than counties with the best air quality (significant at α = 0.05 with Bonferroni correction).

We also used multivariable logistic regression to consider how the various demographic dimensions of communities drive associations with air quality. For each pollution metric, a logistic model estimated the probability of a county being in the worst 20% of counties rather than the best 20% of counties. Models were run using national data, and covariates included all the demographic variables (*i.e.*, percent NHB, percent Hispanic, percent under 5 years of age, percent 65 years and older, and percent in poverty), as well as county population and EPA region. Table 2 presents the odds ratios for a change in each covariate equal to the interquartile range (IQR) of the covariate across all U.S. counties.

When controlling for the above-named other demographic variables, we still see a dramatic difference in the percent NHB in counties with the best and worst air quality as measured by all three pollution metrics; moving from the bottom to the top of the IQR on percent NHB is associated with a 36% to 173% increase in the probability of a county being in the worst 20% of graded counties, depending on the air quality metric of interest. Percent in poverty is positively associated with the probability of a county having the worst air quality for both annual and daily PM_2.5_ (p < 0.01 and p < 0.1, respectively); however, the opposite relationship is seen for ozone (p < 0.01). For each pollution metric, one aspect of the age composition is significantly associated with air quality ranking; counties with the worst air quality have a younger age distribution compared to counties with the best air quality.

In each multivariable logistic model, the EPA region covariate was significant (p < 0.05 for both PM_2.5_ models and p = 0.05 for ozone model), which may indicate that the relationship between county demographics and air quality grades plays out differently in different geographic regions. Although we would like to investigate how the communities with best and worst air quality compare within regions using multivariable analysis parallel to that performed nationally, some regions do not have air quality monitoring data for a sufficient numbers of counties to permit such model-fitting.

We note that the odds ratios between the daily PM_2.5_ model and the annual PM_2.5_ design value model are in the same direction but differ in magnitude. This is reasonable given that the former models short term peak exposures, and the latter measures long term exposures, which do not correlate perfectly.

### 3.3. Demographic Characteristics of Proximate Communities

**Figure 3.**
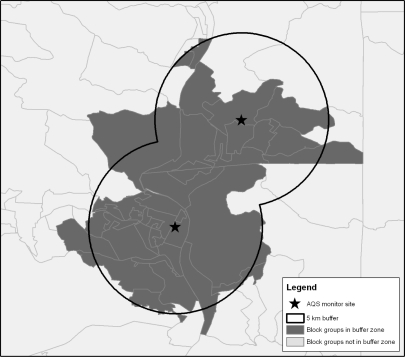
Representation of the area captured by 5 km buffer of AQS monitor sites.

**Figure 4.**
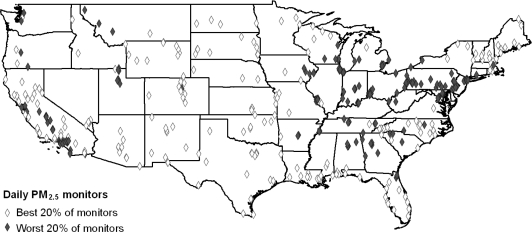
Location of the 20% of monitors with the best air quality and the 20% of monitors with the worst air quality for daily PM_2.5_.

The best/worst analysis described above was executed at the county level. To assess demographic differences at a more refined geographic scale, we selected the 20% of monitors reporting the best air quality and the 20% of monitors reporting the worst air quality. We then created a 5 km buffer around each monitoring site. Figure 3 provides a representation of how the buffers were constructed, and Figure 4 shows the distribution of the cleanest *versus* dirtiest monitors using daily PM_2.5_ as an example (*i.e.*, the map of cleanest *versus* dirtiest monitors is different for ozone). Using a previously developed buffering method [31], Census block groups for which 50% of the area was contained within the 5 km buffer were considered to be within the buffer zone and included in the analysis. Buffer analysis could not be undertaken for annual PM_2.5_ as site-specific data are not available.

Similar to the county-level analysis, we began by exploring the univariate relationships between demographic composition and air quality for communities within the 5 km buffer zone around monitoring sites. For both daily PM_2.5_ and ozone, compared to communities surrounding monitors with the best air quality, those surrounding monitors with the worst air quality are characterized by a higher percent NHB, higher percent under 5 years of age, and lower percent 65 years and older (significant at α = 0.05 with Bonferroni correction). Communities surrounding monitors with the worst air quality in terms of daily PM_2.5_ have higher percent Hispanic and higher percent in poverty than communities around monitors with the best air quality (significant at α = 0.05 with Bonferroni correction). In contrast, the communities surrounding monitors with the worst air quality in terms of daily ozone have lower percent Hispanic and lower percent in poverty compared to communities around monitors with the best air quality (significant at α = 0.05 with Bonferroni correction).

To supplement the univariate analyses, Table 3 summarizes the results of multivariable logistic models for the probability of a Census block group being within the buffer zone of a site ranking in the worst 20% *versus* the best 20% of monitoring sites for each air pollution metric. The logistic model for each pollution metric was constructed similarly to the county-level analysis above and the results are again presented as the odds ratios for a change in each covariate equal to the IQR of the covariate across all U.S. counties. The exception is population, for which the odds ratio was calculated as an increase of 1,000 individuals in order to more reasonably correspond to a plausible change in block group population.

Table 3 provides interesting contrasts to the univariate analysis. In the multivariable logistic models for PM_2.5_, moving from the bottom to the top of the IQR for percent NHB or percent Hispanic, is associated with a 32% and 9%, respectively, increase in the likelihood of being in the worst air quality areas. Age (whether young or old), percent in poverty, and higher population were associated with a decreased risk of being in areas around the 20% of monitors with the worst air quality. For ozone, moving from the bottom to the top of the IQR for percent NHB is associated with a 6% increase in the likelihood of being in the dirtiest air quality areas. However, moving from the bottom to the top of the IQR for percent Hispanic is associated with a 6% decrease in the likelihood of being in the dirtiest air quality areas. Moving from the bottom to the top of the IQR for percent under 5 years of age is associated with a 7% increase in the likelihood of being in the worst ozone areas, whereas older age was associated with a decreased risk of being in the worst 20% of areas, as was percent in poverty. Higher population was associated with an increased risk of being in the worst ozone areas (a 24% increase in risk for each additional 1,000 individuals per block group). Unfortunately, we were unable to perform this analysis disaggregated by EPA region due to the uneven representation of monitors with the best and worst air quality across EPA regions.

### 3.4. Summary of Environmental Justice Results

In both univariate and multivariate analyses, NHB are more likely to live in counties where particulate matter and ozone are well-monitored, but, among monitored locations, they are much more likely to live in areas with the worst air quality. In univariate analyses, Hispanics are also more likely to live in counties where particulate matter and ozone are well-monitored and are more likely to live in areas with the worst air quality. The latter result loses significance in the multivariate analyses

Poverty rates do not differ between non-monitored counties for particulate matter *versus* monitored counties. Non-monitored counties for ozone are characterized by higher rates of poverty. In monitored counties, multivariate analysis suggests that counties with the worst particulate matter air quality are characterized by higher rates of poverty. In contrast, counties with the worst ozone air quality are characterized by lower rates of poverty. This may be due to the broader geographic scale at which high ozone levels tend to present.

In areas immediately proximate to the monitors, NHB are more likely to live in areas with the worst daily PM_2.5_ and ozone air quality. Hispanics are more likely to live in areas with the worst daily PM_2.5_ air quality, but less likely to live in areas with the worst ozone levels. The areas proximate monitors recording the worst daily PM_2.5_ and ozone levels are characterized by lower rates of poverty.

Taken together, these results suggest that NHB in the United States suffer worse air quality across multiple metrics, geographic scales, and multiple pollution metrics. Hispanics also suffer worse air quality with respect to particulate matter, but not necessarily so for ozone. It also appears that environmental justice concerns are more prominent along race/ethnicity lines, rather than measures of poverty.

## 4. Conclusions

This paper provides an analysis of how the Clean Air Act and its Amendments have shaped air quality in both advantaged and disadvantaged communities in the United States. The results suggest, first, that the placement of monitors across the United States emphasizes more urban and densely populated communities. This means that rural areas, which are generally characterized by older, non-Hispanic white populations, are less likely to be monitored. It also means that non-Hispanic blacks and Hispanics are more likely to have access to monitoring data. Consequently, in areas without monitors, researchers, community members, and policy makers all lack access to information about local air quality. Second, we find that counties with the worst PM_2.5_ air quality are characterized by a statistically significant larger percent of NHB, smaller percent of people over 64 years of age, larger percent of people in poverty, and, for daily PM_2.5_ only, more people per county. We also find that counties with the worst ozone air quality are characterized by a statistically significant larger percent NHB, larger percent children under 5 years of age, smaller percent in poverty, and larger populations. Third, using buffering analysis to analyze at a more refined geographic scale, we found significant relationships between race, age, poverty, and air quality for both PM_2.5_ and ozone. Taken together, these results suggest that air quality is uneven across different demographic groups in the United States.

## Figures and Tables

**Table 1.** Mean demographic composition of U.S. counties with and without sufficient monitoring data to receive an ALA air quality grade.

**Demographic**	**EPA region**	**Annual PM_2.5_ (design value)**			**Daily PM_2.5_**			**Ozone**		

		**Suff monitoring**	**Insuff monitoring**		**Suff monitoring**	**Insuff monitoring**		**Suff monitoring**	**Insuff monitoring**	
% non-Hispanic Black	US	13.7	9.0	*	13.6	8.2	*	12.2	11.4	
	1	6.0	2.1		5.6	1.7		5.2	1.8	
	2	17.3	6.2	*	16.4	5.8	*	11.9	18.1	
	3	18.2	14.3		18.9	11.5		15.9	17.4	
	4	22.1	17.8		22.1	17.6		21.0	19.4	
	5	15.6	2.2	*	15.2	2.2	*	14.4	2.9	*
	6	15.6	11.9		16.8	10.0		13.6	13.3	
	7	11.6	2.2	*	11.5	2.1	*	10.7	4.1	
	8	2.7	0.5	*	2.6	0.5	*	2.8	0.5	
	9	6.0	3.9		6.0	1.5	*	5.9	0.9	*
	10	4.0	0.8	*	3.4	0.7	*	3.4	1.1	

% Hispanic	US	15.5	7.4	*	15.1	6.8	*	15.1	6.7	*
	1	7.9	2.7	*	7.4	1.8	*	6.9	1.7	*
	2	18.1	5.4	*	17.3	4.4	*	14.9	13.9	
	3	4.1	3.3		4.6	2.1	*	4.6	1.9	*
	4	9.8	3.3	*	9.5	3.4	*	9.8	3.1	*
	5	6.8	2.1	*	6.7	2.1	*	6.4	2.4	*
	6	27.9	20.1		27.2	19.5		28.6	15.4	*
	7	4.1	3.3		4.2	3.2		3.6	3.8	
	8	12.8	7.1		12.4	7.2		12.8	7.2	
	9	31.5	17.3	*	30.9	17.3	*	30.8	12.7	*
	10	5.7	9.0		5.9	9.7		5.9	9.1	

% under 5 years of age	US	6.9	6.6	*	6.9	6.5	*	6.9	6.5	*
	1	6.3	5.9		6.3	5.7	*	6.2	5.9	
	2	6.6	6.3		6.6	6.2		6.6	6.4	
	3	6.3	6.1		6.3	6.0		6.3	6.0	
	4	6.5	6.5		6.5	6.5		6.5	6.6	
	5	6.9	6.5	*	6.9	6.4	*	6.9	6.4	*
	6	7.8	7.2	*	7.7	7.1	*	7.8	6.9	*
	7	7.0	6.4	*	7.0	6.3	*	6.9	6.5	*
	8	7.6	6.7		7.7	6.5	*	7.8	6.4	*
	9	7.3	6.6	*	7.3	6.4	*	7.3	6.3	*
	10	6.5	7.0		6.7	6.8		6.6	7.0	

% over 64 years of age	US	11.9	13.3	*	11.9	13.5	*	12.0	13.5	*
	1	13.4	13.9		13.4	14.4		13.6	13.4	
	2	13.0	13.0		12.9	13.3		13.1	12.9	
	3	13.1	14.0		13.3	13.9		13.3	14.0	
	4	13.3	13.8		13.3	13.9		13.5	13.4	
	5	12.0	13.6	*	12.0	13.6	*	12.0	13.7	*
	6	9.7	11.9	*	9.8	12.1	*	9.4	13.2	*
	7	12.0	15.5	*	12.1	15.6	*	11.8	15.2	*
	8	9.6	12.3	*	9.4	13.0	*	8.9	13.5	*
	9	10.8	13.0	*	10.8	14.4	*	10.9	14.5	*
	10	10.7	12.0		10.5	12.7		11.1	11.7	

% in poverty	US	12.4	12.4		12.4	12.5		11.8	13.7	*
	1	10.0	7.2		9.5	7.6		9.1	9.1	
	2	14.3	8.7		13.6	9.4		11.5	14.9	
	3	9.6	12.2		10.4	11.6		9.6	13.5	*
	4	13.0	14.9	*	13.0	15.1	*	12.6	15.5	*
	5	10.7	8.8	*	10.6	8.9	*	10.2	9.6	
	6	16.0	16.2		16.5	15.6		15.3	17.4	
	7	9.5	11.5		9.5	11.6		9.1	11.6	
	8	9.9	11.4		9.6	12.1	*	8.8	13.2	*
	9	13.9	14.3		13.9	14.3		13.9	15.6	
	10	10.1	11.8		9.8	12.7	*	10.1	11.9	

* Population-weighted t-test significant at α = 0.05 with Bonferroni correction for multiple comparisons.

**Table 2.** Multivariable logistic regressions modeling the probability of an ALA-graded U.S. county being in the worst 20% of counties *versus* the best 20% of counties for each air pollution metric^a^.

	**Annual PM_2.5_**	**Daily PM_2.5_**	**Ozone**
% non-Hispanic black	2.73 **	1.58 *	1.36 *
% Hispanic	0.83	1.13	0.89
% under 5 years of age	2.09	1.34	1.68 *
% over 64 years of age	0.25 **	0.51 *	0.71
% in poverty	3.95 ***	1.92 *	0.44 ***
Population in 100,000s	1.01	1.19 ***	1.12 ***
R-squared	0.60	0.51	0.34

a Values reported as odds ratios for a change equal to the IQR for each demographic variable based on all U.S. counties. Note: EPA region was also included as a covariate in all models. The Type III p-value for the EPA region covariate was <0.05 in both PM_2.5_ models and 0.052 in the ozone model.

* p < 0.1

** p < 0.05

*** p < 0.01.

**Table 3.** Multivariable logistic regressions modeling the probability of a Census blockgroup being within the 5 km buffer zone of the dirtiest 20% of monitoring sites *versus* cleanest 20% of monitoring sites for each air pollution metric^a^.

	
	**Daily PM_2.5_**	**Ozone**

% non-Hispanic black	1.32***	1.06***
% Hispanic	1.09***	0.94***
% under 5 years of age	0.96***	1.07***
% over 64 years of age	0.90***	0.97**
% in poverty	0.94***	0.90***
Population	0.85***	1.24***
R-squared	0.40	0.18	

a Values for population reported as odds ratios for an increase in the population of 1,000. All other values reported as odds ratios for a change equal to the IQR for each demographic variable based on all U.S. counties. Note: EPA region was also included as a covariate in both models. The Type III p-value for the EPA region covariate was <0.01 in both models.

* p < 0.1

** p < 0.05

*** p < 0.01.
